# Potential Biomarkers of Fatigue Identified by Plasma Metabolome Analysis in Rats

**DOI:** 10.1371/journal.pone.0120106

**Published:** 2015-03-20

**Authors:** Satoshi Kume, Masanori Yamato, Yasuhisa Tamura, Guanghua Jin, Masayuki Nakano, Yukiharu Miyashige, Asami Eguchi, Yoshiyuki Ogata, Nobuhito Goda, Kazuhiro Iwai, Emi Yamano, Yasuyoshi Watanabe, Tomoyoshi Soga, Yosky Kataoka

**Affiliations:** 1 Cellular Function Imaging Team, Division of Bio-function Dynamics Imaging, RIKEN Center for Life Science Technologies, Kobe, Hyogo, Japan; 2 Department of Physiology, Osaka City University Graduate School of Medicine, Osaka, Japan; 3 Graduate School of Life and Environmental Sciences, Osaka Prefecture University, Osaka, Japan; 4 Department of Life Science and Medical Bio-Science, School of Advanced Science and Engineering, Waseda University, Tokyo, Japan; 5 Department of Molecular and Cellular Physiology, Graduate School of Medicine, Kyoto University, Kyoto, Japan; 6 Pathophysiological and Health Science Team, RIKEN Center for Life Science Technologies, Kobe, Hyogo, Japan; 7 Institute for Advanced Biosciences, Keio University, Tsuruoka, Yamagata, Japan; Mayo Clinic, UNITED STATES

## Abstract

In the present study, prior to the establishment of a method for the clinical diagnosis of chronic fatigue in humans, we validated the utility of plasma metabolomic analysis in a rat model of fatigue using capillary electrophoresis-mass spectrometry (CE-MS). In order to obtain a fatigued animal group, rats were placed in a cage filled with water to a height of 2.2 cm for 5 days. A food-restricted group, in which rats were limited to 10 g/d of food (around 50% of the control group), was also assessed. The food-restricted group exhibited weight reduction similar to that of the fatigued group. CE-MS measurements were performed to evaluate the profile of food intake-dependent metabolic changes, as well as the profile in fatigue loading, resulting in the identification of 48 metabolites in plasma. Multivariate analyses using hierarchical clustering and principal component analysis revealed that the plasma metabolome in the fatigued group showed clear differences from those in the control and food-restricted groups. In the fatigued group, we found distinctive changes in metabolites related to branched-chain amino acid metabolism, urea cycle, and proline metabolism. Specifically, the fatigued group exhibited significant increases in valine, leucine, isoleucine, and 2-oxoisopentanoate, and significant decreases in citrulline and hydroxyproline compared with the control and food-restricted groups. Plasma levels of total nitric oxide were increased in the fatigued group, indicating systemic oxidative stress. Further, plasma metabolites involved in the citrate cycle, such as cis-aconitate and isocitrate, were reduced in the fatigued group. The levels of ATP were significantly decreased in the liver and skeletal muscle, indicative of a deterioration in energy metabolism in these organs. Thus, this comprehensive metabolic analysis furthered our understanding of the pathophysiology of fatigue, and identified potential diagnostic biomarkers based on fatigue pathophysiology.

## Introduction

Fatigue is best defined as difficulty in initiating or sustaining voluntary activities [1] and is thought to be accompanied by deterioration of performance [2]. Half of the general population in modern society currently experiences chronic and complex (a combination of physical and mental) fatigue caused by continual stress and prolonged deficiency of rest or sleep [3]. Besides the chronic and complex fatigue seen in our daily life, there are patients with chronic fatigue syndrome (CFS) exhibiting persistent fatigue which is substantially unrelieved by rest, and accompanied by other symptoms such as circadian rhythm sleep disorder and cognitive dysfunction for a minimum of 6 months [4]. CFS is thought to have a worldwide prevalence of 0.4–1.0%. The pathogenesis of CFS remains incompletely understood, and the objective diagnostics based on the pathophysiology remains to be developed.

Metabolic alterations induced by fatigue have been previously reported. After exhaustive or sustained exercise in humans, blood levels of branched-chain amino acids (BCAAs) are decreased compared to pre-exercise levels, whereas free tryptophan levels are increased [5,6]. In addition, mental fatigue loading in humans leads to a decrease in blood levels of the BCAAs, tyrosine, cysteine, methionine, lysine, and arginine [7].

In order to investigate the pathophysiology of fatigue in multiple organs, we have established a complex fatigue animal model where rats are exposed to relatively long-lasting stress and partial sleep deprivation, which humans often experience in their daily lives [8]. In this model, rats were housed in a cage filled with water to a depth of 2.2 cm for 5 days. The fatigued rats showed a significant decrease in swimming time in a weight-loaded forced swimming test, indicative of fatigue [2,8]. The fatigued rats exhibited dysfunction in multiple systems, including the neuro-immuno-endocrine system; specifically, they exhibited increased turnover of 5-hydroxytryptamine in the central nervous system [8], chronic up-regulation of gene expression for the precursor peptides of adrenocorticotropin, β-endorphin, and α-melanocyte stimulating hormone (α-MSH) in the pituitary gland [9], an increase in the peripheral blood level of α-MSH released from the intermediate lobe of hypophysis [10], and an accumulation and an activation of microglial cells in the dorsal horn of spinal cord [11]. Furthermore, levels of BCAAs, such as valine, leucine, and isoleucine, were increased in the plasma, skeletal muscle, liver, and brain, whereas levels of other amino acids, such as glutamine, phenylalanine, tyrosine, arginine, and threonine, were decreased in fatigued rats [2]. The decrease in plasma arginine suggested the facilitation of nitric oxide (NO) production by NO synthase in fatigued rats. Rats experiencing food restriction, which show a weight reduction comparable to fatigued rats, exhibited a different amino acid profile from that of fatigued rats [2]. These findings indicated that the pathophysiology of fatigue includes abnormalities in the neuro-immuno-endocrine system, as well as in metabolism. Although those reports have implied that blood amino acids levels could serve as biomarkers for the diagnosis of physical and/or mental fatigue, more comprehensive metabolite profiling is expected to result in the identification of optimal biomarkers based on the pathophysiology of fatigue.

Recently, metabolome analysis has become a rapidly evolving analytical technology for the comprehensive identification and quantification of endogenous metabolites in samples collected from blood or tissues [12]. This method enables the identification of metabolites or metabolic pathways involved in disease pathophysiology. The efficacy of this experimental approach was demonstrated by the discovery of biomarkers for aging [13], drug–induced gastric injury [14], dilated cardiomyopathy [15], and type 2 diabetes [16].

In the present study, prior to engaging in clinical trials using metabolomic analysis to identify diagnostic biomarkers of chronic fatigue in humans, we conducted a plasma metabolomic analysis of a rat model of fatigue using capillary electrophoresis-mass spectrometry (CE-MS). The objective of this study was to evaluate the utility of a comprehensive analysis of metabolites for identifying potential diagnostic biomarkers of chronic fatigue. In order to identify food intake-independent metabolic changes, plasma samples from fatigued animals were compared to those obtained from a food-restricted group, which exhibited a weight reduction similar to that of the fatigued group. Metabolites potently influenced by food restriction were eliminated from the biomarker candidates for chronic fatigue. Multivariate analyses were employed for determination of potential biomarkers reflecting the pathophysiology of fatigue. Furthermore, we investigated adenosine triphosphate (ATP) levels in organs and erythrocytes, as well as the levels of plasma NO, which are closely associated with metabolic profiles.

## Materials and Methods

### Study design for a fatigue-loaded animal model

Eight-week old male Sprague–Dawley rats (Shizuoka Laboratory Animal Cooperative; Shizuoka, Japan) were used in this study. The animals were housed in a cage with a raised mesh base under constant environmental conditions (room temperature, 22–23°C; relative humidity, 50%-60%) and a 12-h light-dark cycle (08:00/20:00). Prior to the experiment, food and water were provided *ad libitum*. All experimental protocols were approved by the Ethics Committee on Animal Care and Use of the RIKEN Center for Life Science Technologies (MAH19–01–13) and were performed in accordance with the Principles of Laboratory Animal Care (NIH publication No. 85–23, revised 1985). All efforts were made to minimize animal suffering and the number of animals used for the studies.

Rats were randomly divided into three groups; control, food-restriction, and fatigued groups. In order to produce an animal model of relatively long-lasting fatigue, rats underwent deprivation of rest and sleep by being housed in a cage filled with water (23±1°C) to a height of 2.2 cm for 5 days, as previously reported by Jin et al [2]. Because the rats exhibited a reduction in body weight during the fatigue period, we included a food-restricted group that produced a degree of weight reduction similar to the fatigued rats. The food-restricted group was housed in normal cages for 5 days and their food intake was restricted to 10 g/day, as described previously [2]. A non-treated control group was also included.

### Plasma and tissue preparations

Rats were deeply anaesthetized with intraperitoneal injections of sodium pentobarbital (100 mg/kg), and whole blood samples were obtained from the abdominal aorta. The brain (cerebral cortex), liver, and skeletal muscle were removed after perfusion with ice-cold phosphate buffered saline. The blood samples (1.5 ml) were collected into tubes containing 35 μl of 0.06 g/ml (w/v) EDTA and plasma was obtained following centrifugation at 1000 × g at 4°C for 10 min.

### Capillary electrophoresis time-of-flight mass spectrometry (CE-TOFMS) analysis

For extraction of plasma metabolites, 50 μl plasma samples were vortexed after adding 450 μl of methanol containing internal standards (20 μM each of L-methionine sulfone, D-Camphol-10-sulfonic acid and 2-morpholinoethanesulfonic acid). The samples were then mixed with 200 μl Milli-Q water and 500 μl chloroform and centrifuged at 4,600 × g at 4°C for 5 min. Subsequently, the aqueous solution was centrifugally filtered through a 5-kDa cutoff filter (Millipore) at 9,100 × g at 4°C for 3h. The filtered samples (300 μl) were centrifugally concentrated at 35°C for 2h. The dried samples were dissolved in 25 μl Milli-Q water containing reference compounds (200 μM each of 3-aminopyrrolidine and trimesate) for capillary electrophoresis time-of-flight mass spectrometry (CE-TOFMS) analysis [17,18].

CE-TOFMS analysis was performed using an Agilent CE capillary electrophoresis system (Agilent Technologies, Waldbronn, Germany), an Agilent G3250AA liquid chromatography/mass selective detector time-of-flight system (LC/MSD TOF; Agilent Technologies, Palo Alto, CA, USA), an Agilent 1100 series binary high-performance liquid chromatography (HPLC) pump with a G1603A Agilent CE-MS adapter and a G1607A Agilent capillary electrophoresis electrospray ionization mass spectrometry (CE-ESI-MS) sprayer kit. For anion analysis, an Agilent G710060041 platinum ESI needle was used. G2201AA Agilent ChemStation software for CE and Analyst QS Agilent TOFMS software were used for system control and data acquisition. Capillary electrophoresis tandem mass spectrometry (CE-MS/MS) analyses for compound identification were performed on a QStar XL Hybrid liquid chromatography tandem mass spectrometry system (LC-MS/MS; Applied Biosystems, Foster City, CA. USA) connected to an Agilent CE instrument.

### CE-TOFMS conditions for cationic metabolite analysis

Separations were carried out in a fused silica capillary (50 mm internal diameter and 100 cm total length) filled with 1 M formic acid as the electrolyte [19–21]. Approximately 3 nl of sample solution were injected at 50 mbar for 3 sec, and a 30 kV voltage was applied. The capillary temperature was maintained at 20°C and the sample tray was cooled below 5°C. Methanol/water (50% v/v) containing 0.1 μM Hexakis (2,2-difluoroethoxy)phosphazene was delivered as the sheath liquid at 10 μl/min. CE-ESI-TOFMS was operated in the positive ion mode and the capillary voltage was set at 4 kV. A flow rate of heated dry nitrogen gas (heater temperature, 300°C) was maintained at 10 psig. In TOFMS, the fragmentor, skimmer, and octapole radio frequency voltages (Oct RFV) were set at 75V, 50V, and 125V, respectively. Automatic recalibration of each acquired spectrum was achieved using the masses of reference standards ([^13^C isotopic ion of a protonated methanol dimer (2MeOH+H)]^+^, m/z 66.0631; and [Hexakis (2,2-difluoroethoxy)phosphazene + H]^+^, m/z 622.0290). Exact mass data were acquired at a rate of 1.5 spectra/sec over a 50–1,000 m/z range.

### CE-TOFMS conditions for anionic metabolite analysis

A commercially available COSMO(+), chemically coated with cationic polymer, capillary (50 mm internal diameter and 110 cm total length; Nacalai Tesque, Kyoto, Japan) was used as the separation capillary [22]. A 50 mM ammonium acetate solution (pH 8.5) was used as the electrolyte solution for CE separation. Sample solutions (30 nl) were injected at 50 mbar for 30 sec, and a 30 kV voltage was applied. Methanol/water (50% v/v) containing 5 mM ammonium acetate and 0.1 μM Hexakis was delivered as the sheath liquid at 10 μl/min. ESI-TOFMS was conducted in the negative ion mode with the capillary voltage set to 3.5 kV. For TOFMS, the fragmentor, skimmer, and Oct RFV voltages were set at 100V, 50V, and 200V, respectively. Automatic recalibration of each acquired spectrum was performed using the masses of reference standards ([^13^C isotopic ion of deprotonated acetic acid dimer (2CH_3_COOH-H)]^–^, m/z 120.0383; and [Hexakis + deprotonated acetic acid (CH_3_COOH-H)]^–^, m/z 680.0355). Exact mass data were acquired at a rate of 1.5 spectra/sec over a 50–1,000 m/z range. Other conditions were identical to those used in cationic metabolite analysis.

### Measurement of adenosine triphosphate (ATP)

ATP levels of samples were measured using a firefly bioluminescence assay kit (AMERIC-ATP kit; Wako Pure Chemical Industries, Osaka, Japan) [23]. The tissues (brain, liver, and skeletal muscle) and erythrocytes were homogenized immediately in phenol (3 ml) after isolation. The extracted sample (1 ml) was shaken with chloroform (200 μl) and centrifuged at 13,800 × g at 4°C for 5 min. The upper aqueous phase was diluted 2,000-fold for the brain and liver and 10,000-fold for erythrocytes and skeletal muscle with distilled water. The diluted extract (10 μl) was then injected into 90 μl of a luciferin/luciferase mixture and the bioluminescence product was immediately measured using a luminometer (GloMax-96 Microplate Luminometer; Promega, Tokyo, Japan). ATP levels in each sample were calculated from a calibration curve made with ATP standards (0–10^–7^ M).

### Measurement of Nitric Oxide (NO) in Plasma

Nitric oxide production (total concentrations of nitrate and nitrite) was measured using a QuantiChrom Nitric Oxide Assay Kit (Bioassay Systems, Hayward, CA, USA), according to the Griess method [24,25]. A sodium nitrite standard curve was prepared (0–150 μM). Deproteination of the plasma was accomplished by mixing 150 μl of plasma with 8 μl ZnSO_4_, followed by mixing with 8 μl NaOH, vortexing, and centrifuging at 12,000 × g at 4°C for 10 min. Subsequently, 100 μl of each supernatant and the standard were added to 200 μl of the working reagent and incubated at 37°C for 60 min. After centrifugation of the reacted samples at 6,200 × g at 4°C for 5 min, the supernatant was diluted 2-fold with distilled water. Optical density was measured at 550 nm using an Eppendorf BioPhotometer Plus (Eppendorf, Hamburg, Germany). The total nitrite concentrations (μM) were determined according to the sodium nitrite standard curve.

### Data processing, bioinformatics, and Statistical analysis

For data processing of the metabolomic data, missing values were imputed with the lower limit of detection for a given metabolite. The data were normalized using the auto-scaling method (mean-centering and variance-scaling).

For data resampling, outliers in each group were identified using a one-sample t-test. The threshold of data removal was set to *P*-values less than 0.01. The metabolomic data were analyzed with principal component (PC) analysis, and the scores of the primary component of variance (PC1) in each group were tested using the one sample t-test. In this analysis, no outliers were detected. In the ATP and NO data, outliers in the data were identified using a one-sample t-test and removed prior to statistical processing.

Multivariate analyses based on the hierarchical clustering heatmap analysis, PC analysis (PCA), and random forest analysis were performed using the *R* Packages for Metabolomics Univariate and Multivariate Statistical Analysis [26], randomForest, and gplots on the *R* statistics platform (*R* Foundation for Statistical Computing, http://www.r-project.org) [27]. Visualizations of metabolic profiles were performed using VANTED version 2.0.1 [28].

Statistically significant differences were evaluated using a one-way analysis of variance (ANOVA) and Tukey’s honestly significant difference (HSD) post hoc test for comparisons of multiple groups using the *R* statistics package [27] and MetaboAnalyst 2.0 web portal (http://www.metaboanalyst.ca/MetaboAnalyst/) [29,30]. *P* < 0.05 and a false discovery rate (FDR) < 0.1 were considered statistically significant.

## Results

### Profiling of plasma metabolites

**Fig 1:**
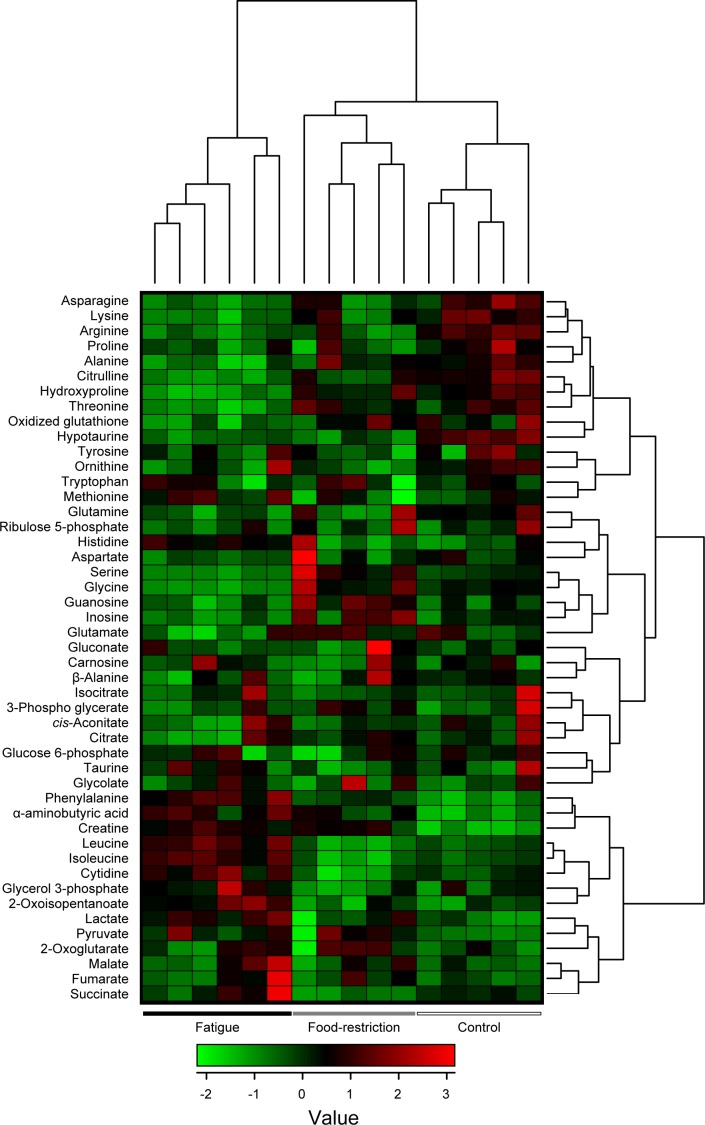
Two-way hierarchical clustering heatmap of plasma metabolome data. Each column shows the metabolic pattern of individual animals in the control group (*n* = 5), food-restricted group (*n* = 5), and fatigued group (*n* = 6). The amount of each metabolite in individual samples is expressed as relative value obtained by the auto-scaling method and is represented by the color scheme in which red and blue indicate high and low concentrations of metabolites, respectively.

**Fig 2:**
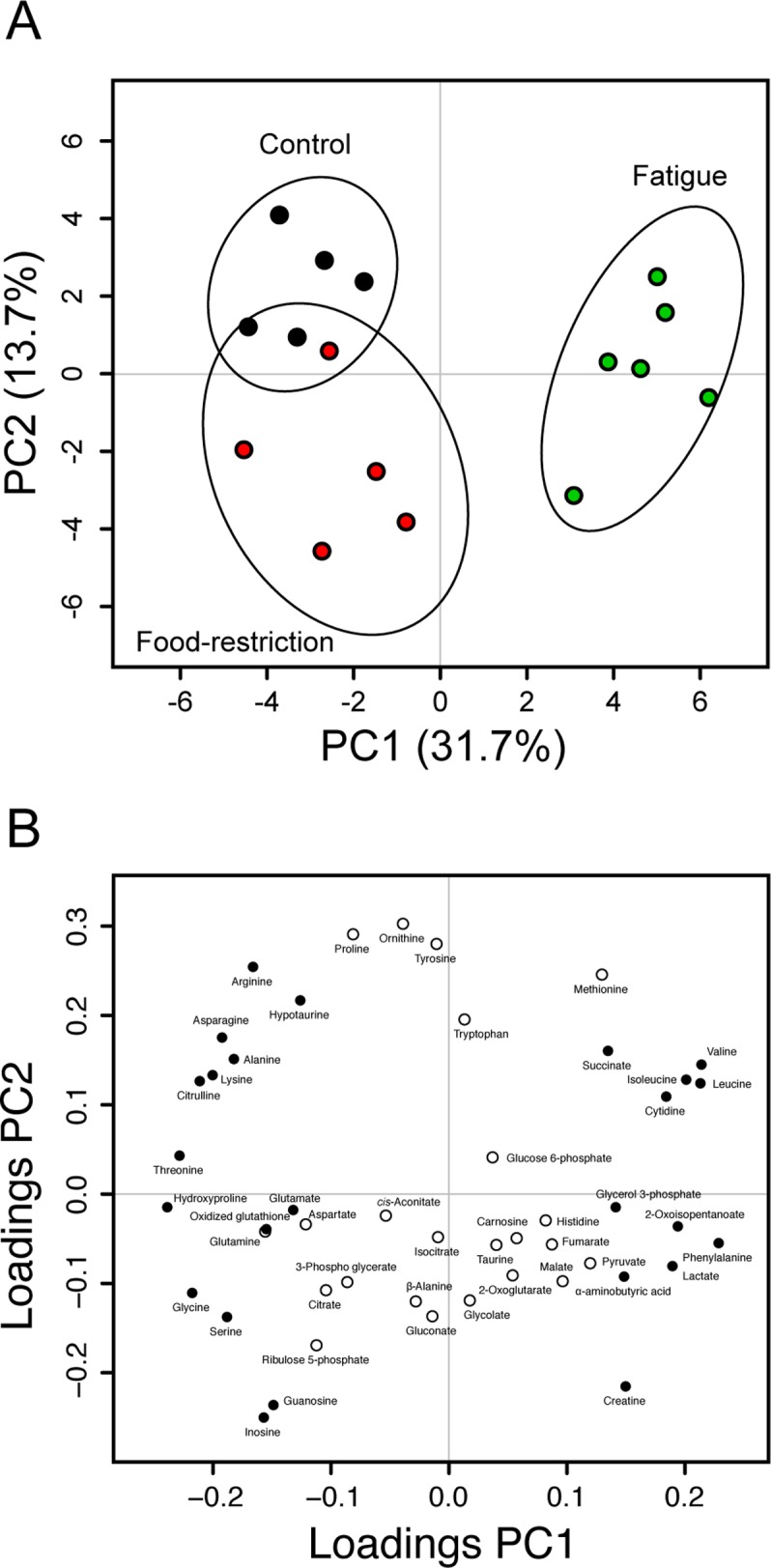
Score and loading plots of principal component analysis of plasma metabolome CE-MS data. (A) PCA score plot of PC2 versus PC1. The control group (*n* = 5), food-restricted group (*n* = 5), and fatigued group (*n* = 5) are shown as black, green, and red circles, respectively. Black ellipses represent the 90% confidence intervals for each group. (B) PCA loading plot of PC2 versus PC1. The data were analyzed after being mean-centered and variance scaled.

The plasma samples in control, food-restricted, and fatigued rats were analyzed with the CE-TOFMS system, and 48 different cationic and anionic metabolites were identified and quantified (Tables 1 and S1). Hierarchical clustering heatmap analysis and principal component (PC) analysis showed characteristic patterns of expression for the metabolites (Figs. 1 and 2). The hierarchical clustering analysis identified three clusters corresponding to the control, food-restricted, and fatigued animal groups. The two-dimensional PCA plot (PC1 vs. PC2) accounted for 45.4% of the total variance (Fig. 2A). The PCA plot revealed that a clear difference was observed in the metabolome profile, indicating that the PC1 axis mainly comprises the variance information of the fatigued group against both the control and food-restricted groups (Fig. 2A). The PC2 axis contained the variance information of the control against the food-restricted group (Fig. 2A). In the PCA loading plot, phenylalanine, valine, leucine, and isoleucine positively contributed to the loadings of PC1, and hydroxyproline, threonine, glycine, and citrulline negatively contributed (Fig. 2B); while ornithine, proline, and tyrosine positively contributed to the loadings of PC2, and inosine, guanosine, and creatine negatively contributed (Fig. 2B).

**Table 1:** Plasma metabolite levels in fatigued animals.

Metabolites (μM)	Control	Food-restriction	Fatigue
2-Oxoglutarate	24.2±3.5	28.2±8.1	27±5.3
2-Oxoisopentanoate	12.7±1.9	12.5±2.6	17.8±2.2^†^ ^§^
α-aminobutyric acid	4.5±0.6	6.4±0.8^†^	7.3±1^†^
3-Phospho glycerate (3PG)	1.2±0.9	1.3±0.3	1±0.4
Alanine	536.2±26.5	509.8±53	434.8±38.4^†^ ^§^
Arginine	333.4±22	249.2±46.8^†^	229.2±22.9^†^
Asparagine	70.1±8.2	60±8.3	53.5±3.1^†^
Aspartate	14.7±1.8	15.2±6.1	12.4±1
β-Alanine	3.5±0.2	3.5±1.2	3.2±0.8
Carnosine	0.8±0.4	0.8±0.6	0.9±0.4
*cis*-Aconitate	5.4±0.8	4.9±0.3	5±1
Citrate	246.6±49.8	246.5±21.7	216.8±56.5
Citrulline	90.6±8.9	75.3±10.1^†^	60.6±5^†^ ^§^
Creatine	169.1±36.8	370.2±51.8^†^	387.4±41.1^†^
Cytidine	8.7±1.2	5.9±2.2	12.4±2.2^†^ ^§^
Fumarate	2±0.3	2.4±0.6	2.6±1
Glucose 6-phosphate (G6P)	2±0.5	1.3±1.1	1.7±1
Glutamine	907.8±62.3	838.8±185.9	752±67.7
Glutamate	93.2±20	104.2±13.8	72.4±21.9^§^
Gluconate	9.4±0.8	10.1±2.8	9.3±1
Glycine	2±0.5	1.9±0.4	1.3±0.3^†^ ^§^
Glycerol 3-phosphate	279.9±25.4	349.8±93.7	160±16^§^
Glycolate	6.5±1.1	5.7±0.9	7.7±1.2
Guanosine	15.3±2.7	16.7±4.9^†^	16±2.5^§^
Histidine	1.1±0.4	2.1±0.5	0.8±0.4
Hydroxyproline	68.1±5.6	69.9±12.4	77.2±2.6^†^ ^§^
Hypotaurine	61.9±10.3	57.7±12^†^	26.4±6.5^†^
Isoleucine	8.3±0.9	3.7±1.2^†^	3.9±0.7^†^ ^§^
Inosine	96.8±8.8	72.4±19.6	144.2±13.5^†^
Isocitrate	14.9±4.3	23.3±8.2	9.8±3
Lactate	6.7±2.4	5.5±0.8	6.4±1.9^†^
Leucine	1972.8±226.2	2148.6±482.5	2637.5±249.5^†^ ^§^
Lysine	180.5±17.4	138±31.2	285.8±31.8^†^
Malate	558.7±65.8	462.9±94.7	369±41
Methionine	15.1±1.7	16.8±4	18.9±5.8
Ornithine	63.3±5.3	56.9±12.2	69.8±7.2
Oxidized glutathione	56.3±4.2	43.9±5.5	48.1±12.4^†^
Phenylalanine	59.9±5.3	72.2±4^†^	89.4±8.2^†^ ^§^
Proline	216.5±30	173.6±36.8	175.1±21.9
Pyruvate	115.4±7.9	166.4±68.2	175.9±40.2
Ribulose 5-phosphate (Ru5P)	7±3.9	7.2±4.3	4.9±2.3
Serine	214.8±17.2	299.8±70^†^	161.1±11.4^§^
Succinate	17.1±1	12.8±1.3	19.7±6^§^
Taurine	124.1±28.5	109.8±14.9	128.2±17.6
Threonine	251.2±36.1	251.8±28.8	175.9±18.3^†^ ^§^
Tryptophan	127.7±6.8	125.6±19.8	125.7±16.3
Tyrosine	96.6±18.5	83.2±10.5	89.1±12
Valine	228.5±18.1	162.2±28.7^†^	352.8±40.3^†^ ^§^

Data are means ± SD (n = 4–6 per group). Rats were kept in normal cage (control), had food restriction (10 g/day, food-restriction) in normal cages, or kept in cages filled with water to the height of 2.2 cm (fatigue) for 5 days.

^†^ *P* < 0.05 and FDR < 0.1, significantly different from the control group (ANOVA followed by Tukey’s HSD post hoc test).

^§^ *P* < 0.05 and FDR < 0.1, significantly different between the food-restricted group and the fatigue group (ANOVA followed by Tukey’s HSD post hoc test).

**Fig 3:**
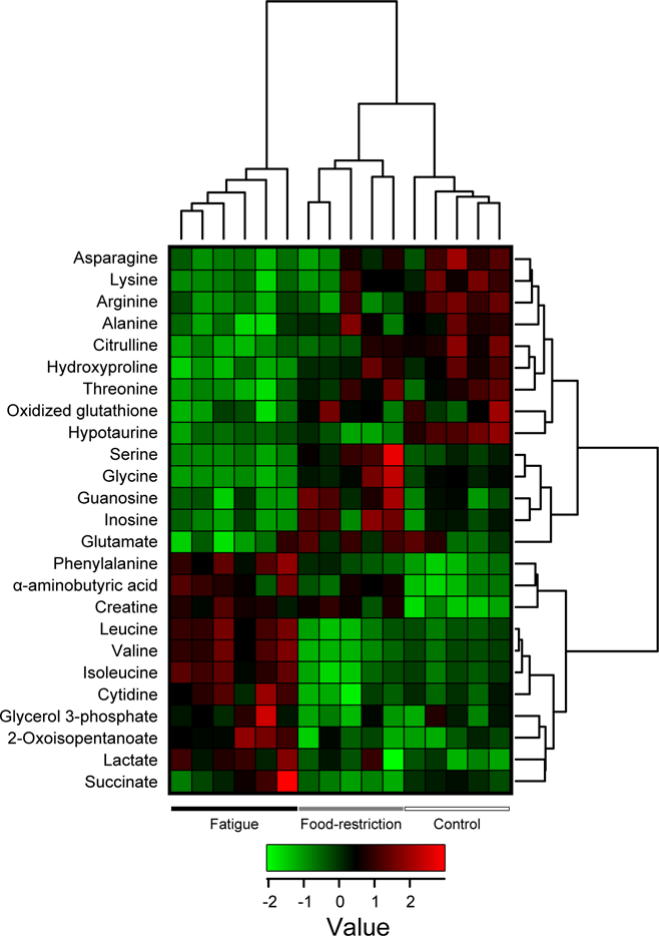
Supervised hierarchical clustered heatmap of 25 metabolites identified by one-way ANOVA. Each column shows the metabolic pattern of individual animals in the control, food-restricted, and fatigued groups. The amount of each metabolite in individual samples is expressed as relative value obtained by the auto-scaling method and is represented by the color scheme in which red and blue indicate high and low concentrations of metabolites, respectively.

**Fig 4:**
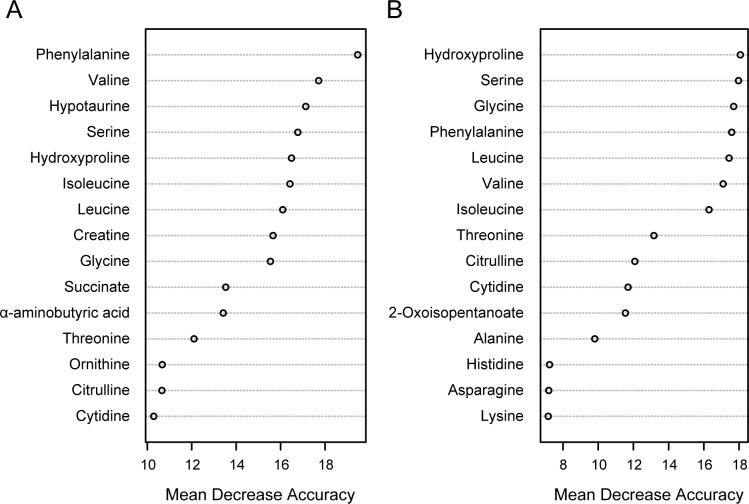
Random forest analysis of the plasma metabolome. The mean decrease in accuracy from the random forest analysis was used to rank metabolites according to their prognostic importance for fatigue status. The 15 most important metabolites among three groups (control, food-restricted, and fatigued) or two groups (fatigued and a combined control and food-restricted group) are shown in A and B, respectively.

Additionally, one-way ANOVA comparison (*P* < 0.05 and FDR < 0.1) identified 25 metabolites that were differentially expressed among the control, food-restricted, and fatigued groups. Hierarchical clustering analysis was performed on the statistically different metabolites, resulting in the demonstration of clear differences among the three treatment groups (Fig. 3). Further, the metabolites were ranked using the mean decrease in accuracy from the random forest analysis (Fig. 4). The most important metabolites for making a discrimination of the fatigued group among three groups (control, food-restricted, and fatigued) contained phenylalanine, valine, hypotaurine, serine, and hydroxyproline (Fig. 4A), while those between two groups (fatigued and a combined control and food-restricted group) contained hydroxyproline, serine, glycine, phenylalanine, and leucine (Fig. 4B). There was a high degree of consistency between the top-scoring metabolites identified by the random forest analysis (Fig. 4) and those identified by hierarchical clustering (Fig. 3). Most of the identified metabolites were closely related to the TCA cycle (succinate), BCAA metabolism (valine, leucine, isoleucine, and 2-oxoisopentanoate), urea cycle (ornithine, citrulline, and arginine), proline metabolism (hydroxyproline), and other amino acid metabolism. These results indicate that fatigue-loading potently influences metabolic profiles in rat plasma.

### Potential biomarkers of fatigue in plasma metabolites

**Fig 5:**
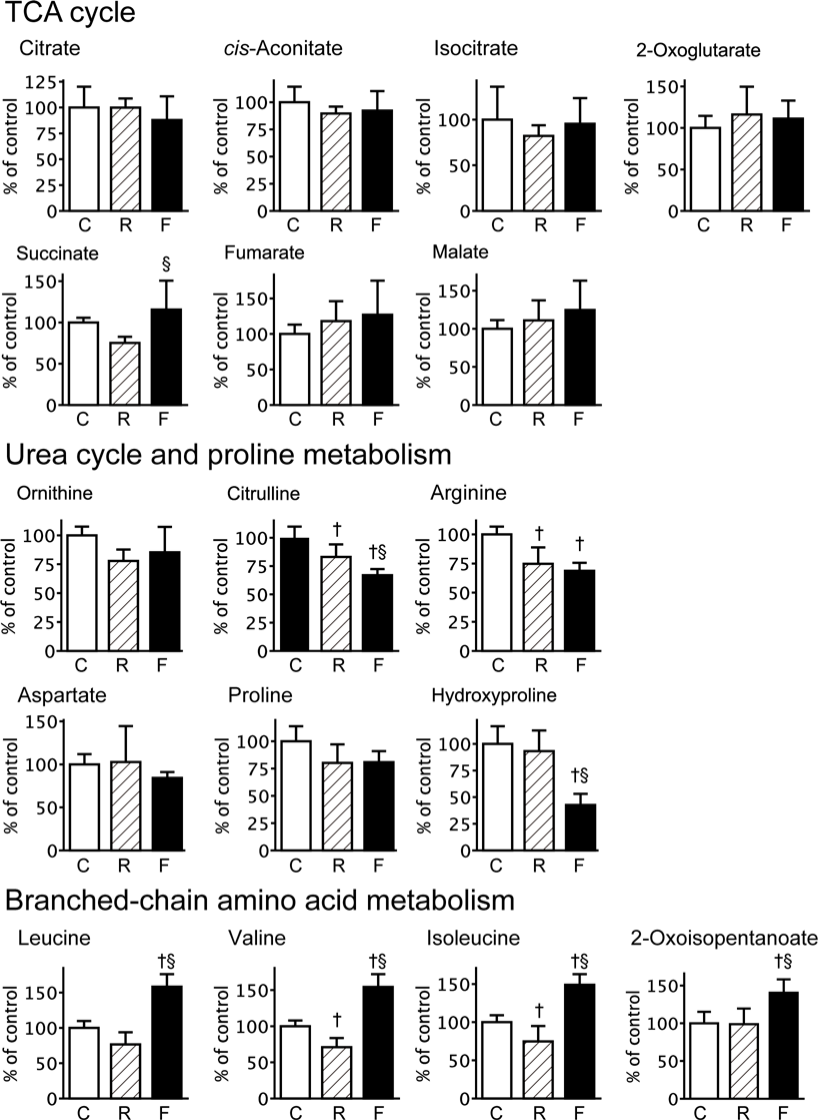
Relative concentrations of plasma metabolites related to the TCA cycle, urea cycle and proline metabolism, and BCAA metabolism. Relative amounts of each metabolite in the control (C, *n* = 5), food-restricted (R, *n* = 5), and fatigued (F, *n* = 6) groups are shown and expressed as a percent of the control group. Data are presented as mean ± S.D. ^†^ *P <* 0.05, significantly different than the control group. ^§^ *P <* 0.05, significantly different between the food-restricted and fatigued group.

In the first steps of the TCA cycle, the levels of organic acids in the fatigued group showed a slight reduction in comparison with those in the control group (citrate decreased by 12.1%, *cis*-aconitate by 7.7%, and isocitrate by 4.5%), while a subset of metabolites in the TCA cycle showed a trend toward overcompensation (2-oxoglutarate increased by 11.2%, succinate by 15.7%, fumarate by 27.0%, and malate by 24.6%) (Fig. 5). The decreases in citrate, *cis*-aconitate and isocitrate in the fatigued group were confirmed by the LC/MS measurements (significant reduction in *cis*-aconitate and isocitrate; S1 Fig and S2 Table). In the food-restricted group, the organic acids of the TCA cycle were slightly changed: *cis*-aconitate decreased by 10.3%, isocitrate by 17.9%, and succinate by 24.7%, while 2-oxoglutarate increased by 16.2%, fumarate by 18.1%, and malate by 11.0% (Fig. 5). Plasma ATP levels in the three groups were below the detection limit of the assay.

The urea cycle is known to counteract the toxic effects of ammonia in the liver. Plasma levels of metabolites related to the urea cycle were reduced in the fatigued group: ornithine decreased by 14.7%, citrulline by 33.1% (*P* < 0.01, compared with the control), and arginine by 31.3% (*P* < 0.01, compared with the control) (Fig. 5). The citrulline levels in the fatigued group were also lower compared to the food-restricted group (Fig. 5). In addition, plasma levels of aspartate and proline in the fatigued group were slightly decreased compared to the control group, while hydroxyproline in the fatigue group was significantly lower than in the control and food-restricted groups (*P* < 0.01) (Fig. 5). Conversely, the fatigued group showed significant increases in the BCAAs valine, leucine, and isoleucine, as well as 2-oxoisopentanoate, compared with those in both the control and food-restricted groups. Thus, the changes in citrulline, hydroxyproline, and BCAAs in the fatigued group were attributed to fatigue-loading, but not to food restriction. In addition, the purine metabolites inosine and guanosine were significantly increased only in the food-restricted group (Table 1).

### Tissue ATP contents

**Fig 6:**
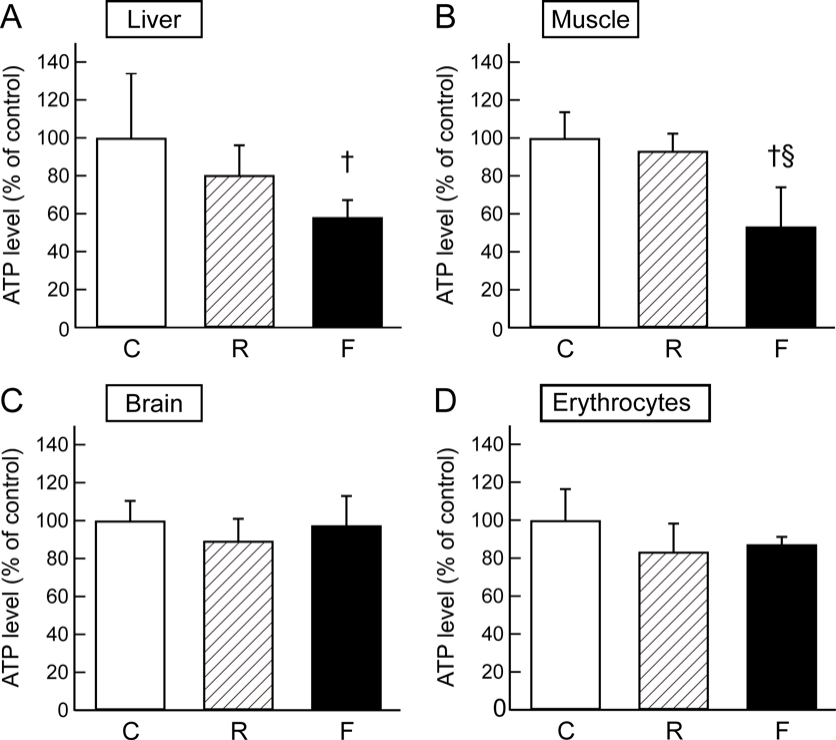
ATP levels in the liver, brain, skeletal muscle, and erythrocytes. Relative levels of ATP in each tissue in the control (C, *n* = 5–6), food-restricted (R, *n* = 5–6), and fatigued (F, *n* = 5–7) groups are shown and expressed as a percent of the control group. Data are presented as mean ± S.D. ^†^ *P <* 0.05, significantly different from the control group. ^§^ *P <* 0.05, significantly different between the food-restricted and fatigued group.

The changes in plasma metabolites involved in the TCA cycle in fatigued rats suggested abnormal energy metabolism during fatigue loading. In order to evaluate energy production and the demand in organs during food restriction or fatigue, we examined tissue ATP levels in the liver, skeletal muscle, brain, and erythrocytes. In the liver, ATP levels in the fatigued group were significantly deceased compared to the control group (*P* = 0.013) (Fig. 6). In the skeletal muscle, ATP levels in the fatigued group showed a significant decrease compared with those in the control (*P* < 0.01) and food restricted groups (*P* < 0.01) (Fig. 6). In the brain and erythrocytes, ATP levels in the fatigued group and food restricted group showed slight reductions compared with that of the control group (Fig. 6). These data indicate that fatigue loading decreases energy production and/or increases energy demand, particularly in the liver and skeletal muscle.

### Fatigue-induced changes in plasma NOx levels

**Fig 7:**
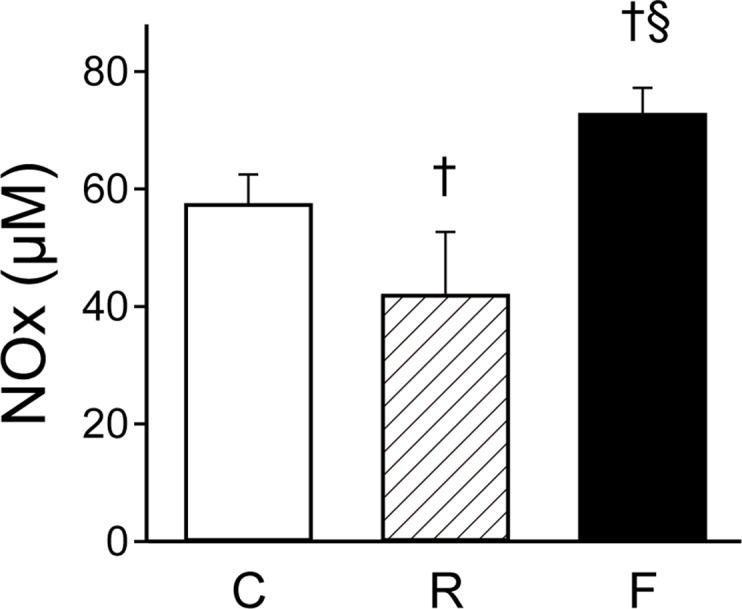
NOx content in the plasma. Plasma NOx content in the control (C, *n* = 5), food-restricted (R, *n* = 6), and fatigued (F, *n* = 6) groups are shown. Data are presented as mean ± S.D. ^†^ *P <* 0.05, significantly different from the control group. ^§^ *P <* 0.05, significantly different between the food-restricted and fatigued group.

In order to assess the relationships between nitric oxide (NO) levels and arginine-citrulline balance under the fatigue condition, we measured total concentrations of nitrate and nitrite (NOx) in plasma as NO levels. The total NOx in the plasma of the fatigued group was significantly increased compared with the control (*P* = 0.01) and food-restricted groups (*P* < 0.01, Fig. 7). Food-restriction induced a significant reduction in NOx levels compared to control (*P* < 0.01, Fig. 7). These results indicate that plasma NO levels are increased under the fatigue condition.

## Discussion

In this study, we performed a comprehensive determination of metabolic alterations in the plasma of fatigued rats. The candidate fatigue biomarkers were differentiated from those affected by alterations in food-intake, and were subjected to multivariate analysis. The metabolomic analysis revealed that fatigue-induced metabolic changes in the plasma were clearly discriminated from those in the non-treated controls and the food-restricted group (Fig. 2). We previously reported fatigue-induced changes in the levels of several amino acids in the plasma of fatigued rats, including increases in BCAAs (valine, leucine, and isoleucine) and phenylalanine, as well as decreases in tryptophan, methionine, lysine, arginine, histidine, serine, threonine, asparagine, aspartate, glutamine, glutamate, glycine, alanine, and proline [2]. As reported by Lehmann et al. [5], plasma levels of many amino acids such as threonine, serine, arginine, proline, glycine, alanine, valine, ornithine, lysine, histidine, and taurine were significantly decreased after the human extreme exercise in the 1993 Colmar ultra triathlon. These systematical decreases in the amino acids are considered to be due to the high consumption of amino acids for compensating a decrease of energy metabolism within a relatively short time. The differences in changing profiles of metabolites between the human extreme exercise and the present rat fatigue model may reflect different in duration and strength of fatigue-load. Recently, Matsui et al. reported that prolonged exhaustive exercise decreased glycogen levels in brain, skeletal muscle, and liver to less than 50% of pre-exercise levels [31]. We identified changes in the plasma metabolites related to the TCA cycle in the fatigued model that trended toward significance (Fig. 5, S1 Fig, and S2 Table), indicating abnormal energy metabolism in several organs. The levels of ATP in liver and skeletal muscle were significantly decreased in the fatigued group (Fig. 6). These results suggested that fatigue influences multiple pathways in primary metabolism, resulting from increased energy demand and decreased supply, along with decreased levels of glycogen and amino acids. The increased energy demand may be correlated to up-regulation of BCAAs (leucine, isoleucine, and valine) due to increased proteolysis in skeletal muscle, as discussed previously [2].

Multivariate analyses using PCA and random forest analysis identified significant decreases in the levels of citrulline and hydroxyproline in the fatigued group, compared with the control and food-restricted groups (Figs. 2 and 4). Citrulline is a component of the urea cycle, is involved in major liver function detoxification, and is produced from ornithine and carbamoyl phosphate by ornithine transcarbamylase [32]. Citrulline is also produced from arginine by the NO production pathway and is catalysed by nitric oxide synthase, which is reported to be up-regulated by AMP-activated protein kinase in response to ADP/ATP imbalance [33], as well as other signalling inputs [34]. The metabolic balance of citrulline and arginine is known to influence intracellular and extracellular lipid peroxidation levels [35,36]. In fatigued animals, plasma NOx levels were observed to be increased (Fig. 7), and plasma oxidative stress levels were also found to be elevated. Indeed, thiobarbituric acid-reactive lipoperoxide was reported to be increased in the liver tissue of fatigued animals [37]. Furthermore, oxidative stress is known to stimulate modifications of amino acids, such as the cleavage and oxidation of proline [38]. The decrease in plasma hydroxyproline observed in the present study might be related to oxidative modifications of proline in the liver. Taken together, chronic fatigue is thought to disturb the balance of citrulline and arginine due to metabolic stress (ADP/ATP imbalance), which leads to oxidative stress, including peroxidation in the liver.

We identified fatigue-induced changes in primary metabolism, including urea cycle, proline metabolism, and BCAA metabolism, in rat plasma. The influence of food-intake and increased proteolysis was taken into consideration, and citrulline and hydroxyproline were newly identified as metabolic signs reflecting complex fatigue in the rat fatigue model. These metabolites may be promising diagnostic biomarkers for human chronic fatigue and/or CFS. As described in Geigy Scientific Tables edited by Lentner, C. [39], some of plasma metabolites such as hydroxyproline are known to show sexual differences in human. If we apply such metabolites to the diagnostic biomarkers in humans, sexually-segregated plasma levels of the metabolites will be needed to be taken into consideration. Taken together, our results may be helpful for realizing the effective prevention and treatment of chronic fatigue by controlling metabolism in humans.

## Supporting Information

S1 Fig Relative concentrations of metabolites related to the TCA cycle in rat plasma. Plasma samples were analyzed using a LC-10A series HPLC (Shimadzu, Kyoto, Japan) equipped with an API 5000 triple quadrupole mass spectrometer (AB Sciex, Foster City, CA, USA). Relative concentrations of TCA cycle metabolites (citrate, *cis*-aconitate, isocitrate, succinate, and malate) in the control group (C, *n* = 3) and fatigued group (F, *n* = 3) are shown and expressed as a percent of the control group. Data are presented as mean ± S.D. ^†^ *P <* 0.05, significantly different from the control group. The LC/MS measurements of rat plasma samples revealed a trend toward decreased citrate (*P* = 0.053) and significant decreases in *cis*-aconitate (*P* = 0.014) and isocitrate (*P* = 0.036) levels.
(TIF)

S1 Table Quantitative metabolome data of rat plasma obtained by CE-MS study. (XLS)

S2 Table Quantitative metabolome data of rat plasma obtained by LC-MS study. (XLS)
