# Supplementary figures and images for: Potential Biomarkers of Fatigue Identified by Plasma Metabolome Analysis in Rats
Source: PLoS One. 2015 Mar 20;10(3):e0120106. doi: 10.1371/journal.pone.0120106 (PMC4368560; doi:10.1371/journal.pone.0120106)

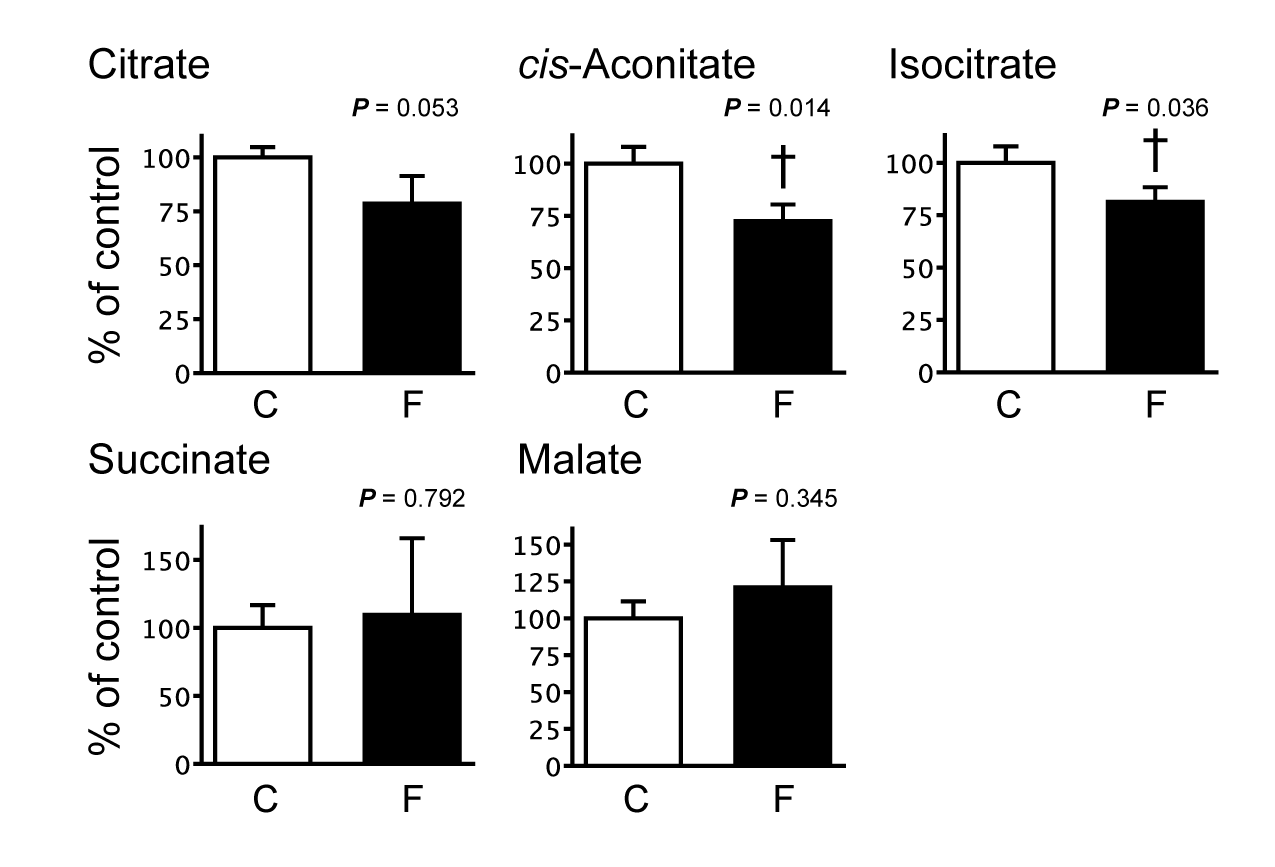

Supplement: S1 Fig — Plasma samples were analyzed using a LC-10A series HPLC (Shimadzu, Kyoto, Japan) equipped with an API 5000 triple quadrupole mass spectrometer (AB Sciex, Foster City, CA, USA). Relative concentrations of TCA cycle metabolites (citrate, cis-aconitate, isocitrate, succinate, and malate) in the control group (C, n = 3) and fatigued group (F, n = 3) are shown and expressed as a percent of the control group. Data are presented as mean ± S.D. † P < 0.05, significantly different from the control group. The LC/MS measurements of rat plasma samples revealed a trend toward decreased citrate (P = 0.053) and significant decreases in cis-aconitate (P = 0.014) and isocitrate (P = 0.036) levels. (TIF) [file pone.0120106.s001.tif]
